# Analysis of Missense Variants in the Human Histamine Receptor Family Reveals Increased Constitutive Activity of E410^6.30×30^K Variant in the Histamine H_1_ Receptor

**DOI:** 10.3390/ijms22073702

**Published:** 2021-04-02

**Authors:** Xiaoyuan Ma, Marta Arimont Segura, Barbara Zarzycka, Henry F. Vischer, Rob Leurs

**Affiliations:** Division of Medicinal Chemistry, Faculty of Science, Amsterdam Institute for Molecules, Medicines and Systems, Vrije Universiteit Amsterdam, 1081 HZ Amsterdam, The Netherlands; x.ma@vu.nl (X.M.); m.arimontsegura@vu.nl (M.A.S.); b.a.zarzycka@vu.nl (B.Z.); h.f.vischer@vu.nl (H.F.V.)

**Keywords:** missense variation, G protein-coupled receptor, histamine H_1_ receptor, ionic lock, constitutive activity

## Abstract

The Exome Aggregation Consortium has collected the protein-encoding DNA sequences of almost 61,000 unrelated humans. Analysis of this dataset for G protein-coupled receptor (GPCR) proteins (available at GPCRdb) revealed a total of 463 naturally occurring genetic missense variations in the histamine receptor family. In this research, we have analyzed the distribution of these missense variations in the four histamine receptor subtypes concerning structural segments and sites important for GPCR function. Four missense variants R127^3.52×52^H, R139^34.57×57^H, R409^6.29×29^H, and E410^6.30×30^K, were selected for the histamine H_1_ receptor (H_1_R) that were hypothesized to affect receptor activity by interfering with the interaction pattern of the highly conserved D(E)RY motif, the so-called ionic lock. The E410^6.30×30^K missense variant displays higher constitutive activity in G protein signaling as compared to wild-type H_1_R, whereas the opposite was observed for R127^3.52×52^H, R139^34.57×57^H, and R409^6.29×29^H. The E410^6.30×30^K missense variant displays a higher affinity for the endogenous agonist histamine than wild-type H_1_R, whereas antagonist affinity was not affected. These data support the hypothesis that the E410^6.30×30^K mutation shifts the equilibrium towards active conformations. The study of these selected missense variants gives additional insight into the structural basis of H_1_R activation and, moreover, highlights that missense variants can result in pharmacologically different behavior as compared to wild-type receptors and should consequently be considered in the drug discovery process.

## 1. Introduction

G protein-coupled receptors (GPCRs) constitute the largest family of membrane proteins encoded by the human genome and play a dominant role in regulating human physiology in response to extracellular stimuli [1]. Consequently, GPCRs are well-established targets for therapeutic intervention with approximately 35% of all United States Food and Drug Administration (USFDA)- and European Medicines Agency (EMA)-approved drugs acting via one or more GPCR subtypes [2,3].

A recent analysis of the protein-coding DNA sequence (exome) of nearly 61,000 unrelated individuals in the exome aggregation consortium (ExAC) database [4] revealed 65,539 missense variations (MV) in the total GPCR family with on average 155.0 rare (single observations or minor allele frequency (MAF) < 1 × 10^−3^) and 4.8 commons (MAF ≥ 1 × 10^−3^) MVs per GPCR subtype (https://gpcrdb.org/mutational_landscape/statistics; accessed on 20 January 2021) (Table 1) [5]. These naturally occurring GPCR variants may display differences in ligand binding, basal activity, signaling, trafficking, and/or expression, as compared to the most frequently occurring wild-type receptor [5]. Hence, individuals in the population might respond differently to medicines due to these genetic variations in their target GPCRs. Indeed, sorting intolerant from tolerant (SIFT; ≤ 0.05) and polymorphism phenotyping (PolyPhen; ≥ 0.1) analyses predicted that 67.1% (9522 of the 14,192) of the MVs identified in 108 GPCR with USFDA-approved drugs in 2017 might affect receptor functioning [5]. Of these MVs, 1722 were found to map to ligand-binding pockets, G protein and/or β-arrestin interaction interface, allosteric sodium-binding pocket, microswitches that are involved in receptor conformational change, and post-translational modification sites [5]. Indeed, variants of the μ-opioid and cholecystokinin-A receptors displayed distinct drug responses in transfected cells [5].

The endogenous biogenic amine histamine is an important regulator in various (patho)physiological processes by acting via the GPCR subfamily of histamine receptors that consist of the histamine H_1_, H_2_, H_3_, and H_4_ receptor (H_1_R, H_2_R, H_3_R, and H_4_R) [6]. The H_1_R and H_2_R are long-known drug targets with antagonists being developed in the 1930s and 1970s, respectively [7]. In fact, the H_1_R has the highest number (73) of approved drugs listed in the “drug-target classification tree” on the GPCRdb website (https://gpcrdb.org/drugs/drugmapping; accessed on 20 January 2021) [3,8]. The majority of these H_1_R drugs are used to relieve allergic reactions by antagonizing H_1_R-mediated contraction of airway smooth muscles and increasing vascular permeability in response to histamine, which is released from mast cells upon allergen binding, but also in the treatment of nausea and vomiting [6]. Moreover, 14 approved drugs are listed in the “GPCRdb drug-target classification tree” for the H_2_R, of which some are “blockbuster” drugs for the treatment of peptic ulcer and gastroesophageal reflux disease by antagonizing H_2_R-mediated gastric acid secretion in response to histamine [6]. The H_3_R and H_4_R were more recently discovered in the 1980s and 2000, respectively [6]. The H_3_R is involved in the regulation of neurotransmission and has been associated with Parkinson’s and Alzheimer’s diseases, epilepsy, learning and sleeping disorders [6]. Although several clinical trials with H_3_R ligands have been reported in the last decade [9], hitherto only pitolisant (Wakix^®^) has been approved in 2016 and 2019 by the EMA and USFDA, respectively, for the treatment of narcolepsy [10,11]. The H_4_R is involved in immunomodulation, and several antagonists are currently in a clinical trial for the treatment of itch, psoriasis, atopic dermatitis, rheumatoid arthritis, allergic rhinitis, and asthma [12].

The ExAC dataset analysis revealed 463 genetic missense variants in the histamine receptor family (Table 1) [5]. In this paper, we first report on the analysis of these genetic variants in the histamine receptor family and provide an experimental example for the H_1_R how these natural variants can contribute to the understanding of GPCR function.

## 2. Results

### 2.1. Genetic Missense Variations in the Histamine Receptor Family

**Figure 1:**
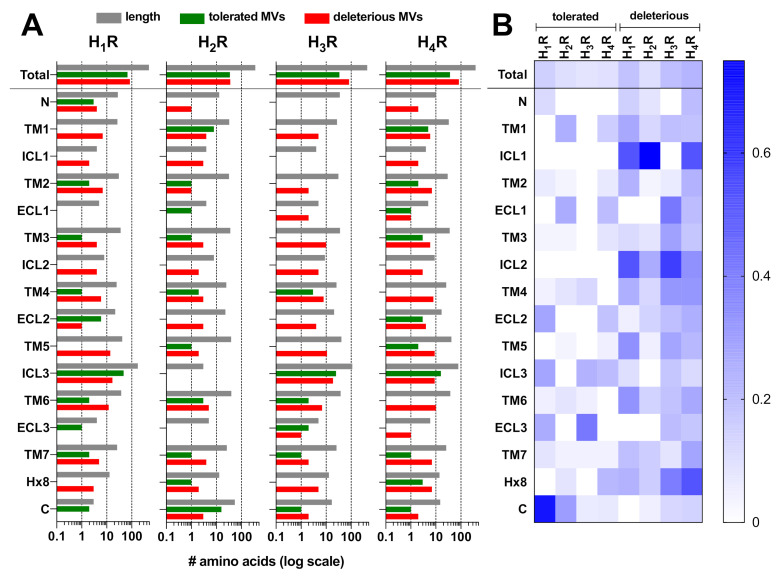
Distribution and predicted impact of 463 genetic missense variants (MVs) in histamine receptor family from nearly 61,000 unrelated individuals. (**A**) The number of tolerated (sorting intolerant from tolerant, SIFT > 0.05 and/or PolyPhen < 0.1) and deleterious (SIFT ≤ 0.05 and/or PolyPhen ≥ 0.1) MVs is plotted in green and red bars, respectively, for the total receptor and for each structural segment. The number of amino acids (length) for each receptor subtype and the individual structural segments is plotted in gray bars. (**B**) MV density was determined by normalizing the number of MVs for the length (i.e., number of amino acids) of each receptor subtype or indicated structural segment. N is N-terminal tail, TM is transmembrane, ICL is intracellular loop, ECL is extracellular loop, Hx8 is helix 8, and C is C-terminal tail.

Analysis of the ExAC dataset revealed 154, 71, 117, and 121 genetic missense variants (MVs) for the H_1_R, H_2_R, H_3_R, and H_4_R, respectively (Figure 1, Table 1) [5]. Most of these 453 MVs are rare with a minor allele frequency (MAF) < 1 × 10^−3^, whereas only 10 MVs are considered common with a MAF score ≥ 1 × 10^−3^ (Table 1). Five common MVs were observed in the N-terminal tail (i.e., M14I, K19N) and ICL3 (i.e., G270E, R326Q, D349H) of H_1_R, one common MV in TM7 (N266^7.31×30^S) of H_2_R, and four common MVs in TM3 (V88^3.26×26^G), TM4(A138^4.48×48^V), TM5 (H206^5.70×70^R) and ICL3 (S284C) of H_4_R (Supplementary Figure S1). Interestingly, approximately half of the MVs in the H_1_R and H_2_R (55.8% and 50.7%, respectively) were predicted to have a functional impact based on their SIFT (0–0.05) and/or PolyPhen (>0.1) scores, whereas more than two-thirds of the MVs in the H_3_R and H_4_R (70.9% and 69.4%, respectively) were predicted to be functionally deleterious [5].

The majority of MVs that are predicted to be tolerated are found in the long intracellular loop (ICL)3 of H_1_R (48 out of 68), H_3_R (25 out of 34), and H_4_R (16 out of 37), whereas H_2_R contains nearly half of the tolerated MVs (16 out of 35) in its relatively long C-terminal tail (Figure 1A, Supplementary Figure S2), which might be consistent with the unstructured nature of these relatively long intracellular domains. However, the long ICL3s of H_1_R, H_3_R, and H_4_R (20–36% of total receptor length) also contain relatively high numbers of potentially deleterious MVs (i.e., 17 out of 86, 19 out of 83, and 9 out of 84, respectively) in comparison to other structural segments (Figure 1A, Supplementary Figure S2). Nonetheless, the majority of deleterious MVs in the histamine receptor family are situated in the transmembrane (TM) and helix 8 domains (i.e., 58 out of 86 for H_1_R; 24 out of 36 for H_2_R; 50 out of 83 for H_3_R; 60 out of 84 for H_4_R), which might not be surprising considering both their size (49–68% of total H_x_R length) and role in transducing extracellular ligand-binding into intracellular G protein signaling via conformational rearrangements. In addition, also the very short ICL1 and ICL2 contain a relatively high-density in deleterious MVs (Figure 1B).

**Figure 2:**
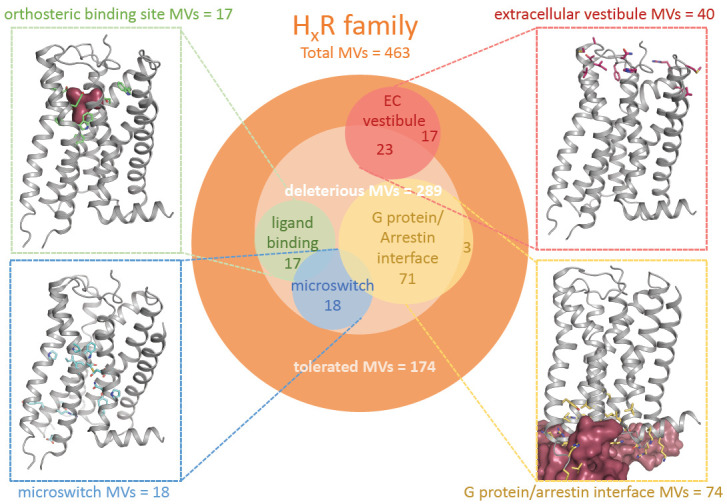
Functional mapping of 463 MVs predicted to be tolerated or deleterious in histamine receptor family from nearly 61,000 unrelated individuals. Tolerated (SIFT > 0.05 and/or PolyPhen < 0.1) and deleterious (SIFT ≤ 0.05 and/or PolyPhen ≥ 0.1) MVs were mapped to functional sites that are known to be involved in histamine receptor-ligand binding (i.e., orthosteric binding site; green), ligand entry trajectory (i.e., extracellular vestibule; red), (in)activation (i.e., microswitches, including the allosteric sodium pocket; blue), and the GPCR-G protein/arrestin-binding interface (gold). The displayed H_1_R structures (Protein Data Bank (PDB) code 3RZE) show MVs in the indicated functional sites.

Moreover, nearly half of the deleterious MVs (129 out of 289) in the histamine receptor family affects key functional GPCR motifs, including the extracellular vestibule involved in ligand entry, the ligand-binding sites, the conserved microswitches involved in GPCR (in)activation (i.e., ionic lock motif, NPxxY motif, CWxP motif, PIF motif, and sodium pocket), and the GPCR-G protein/β-arrestin interaction interface (Figure 2). Indeed, most tolerated MVs (88.5%) are mapped outside these key functional motifs (Figure 2).

**Figure 3:**
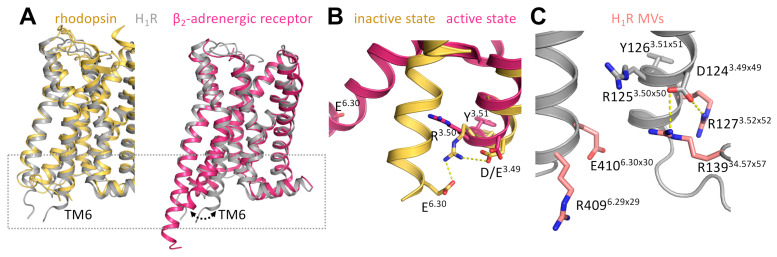
Ionic lock microswitch in inactive and active GPCR structures. (**A**) Overlay of inactive H_1_R (PDB code 3RZE; gray) on inactive rhodopsin (PDB code 1F88; gold) and active β_2_-adrenergic receptor (PDB code 3SN6; magenta) state structures showing the outward movement of TM6 upon receptor activation. (**B**) The ionic lock between E6.30 and R3.50 of the D(E)RY motif in TM6 and TM3, respectively, in inactive rhodopsin (gold), is broken in the active β_2_-adrenergic receptor (magenta) due to the outward movement of TM6. (**C**) Position of the four amino acid residues (pink) with deleterious MVs in TM3 (R127^3.52×52^H), ICL2 (R139^34.57×57^H), and TM6 (R6.39H and E410^6.30×30^K) of the H_1_R structure.

Interestingly, the ExAc dataset reported E410^6.30×30^K in the H_1_R as a rare heterozygous MV with a MAF of 3.30 × 10^−5^ that is predicted to be deleterious by its SIFT/PolyPhen score and functionally maps to both a microswitch and the G protein/arrestin interface (Table 2; Figure 3). The ionic lock between E410^6.30×30^K and the highly conserved D(E)RY motif at the intracellular ends of TM6 and TM3, respectively, in the ground state crystal structure of bovine rhodopsin, has been identified as key interaction to maintain the receptor in an inactive conformation by restricting the distance and orientation between TM3 and TM6 (Figure 3) [13]. Indeed, this distance between TM3 and TM6 is considerably enlarged in the active crystal structure of the β_2_-adrenergic receptor as a consequence of a 14Å outward movement of TM6 (Figure 3) [14]. Importantly, removal of this ionic lock interaction by site-directed mutagenesis increased the constitutive activity of both rhodopsin and β_2_-adrenergic receptor [15,16,17,18], as well as several other GPCRs, including the H_2_R [19]. The ionic lock interaction between R125^3.50×50^ and E410^6.30×30^ is not observed in the inactive crystal structure of doxepin-bound H_1_R (Figure 3), which might be the consequence of substituting ICL3 with the T4-lysozyme for the crystallization process [20]. In fact, this interaction is not always present in crystal structures despite it being proven experimentally [17].

In addition, the ExAc dataset revealed three arginine to histidine MVs in TM3, ICL2, and TM6 of H_1_R that are predicted to be deleterious by their SIFT/PolyPhen scores and situated in the close vicinity of the putative ionic lock and/or at the predicted GPCR-G protein/arrestin interaction interface (Table 2; Figure 3). The H_1_R is known to constitutively activate various G_q_ protein-mediated responses, and consequently, earlier identified H_1_R antagonists were found to actually act as inverse agonists [21,22,23]. We have previously investigated the activation mechanism and constitutive activity of the H_1_R [24,25,26], and therefore, examined the consequence of these four deleterious MVs on H_1_R function. To this end, we introduced these four MVs individually in the wild-type H_1_R and evaluated their effect on ligand binding and G_q_ protein-mediated signaling.

### 2.2. The E410^6.30×30^K Natural Variant Displays Increased Histamine Affinity

**Figure 4:**
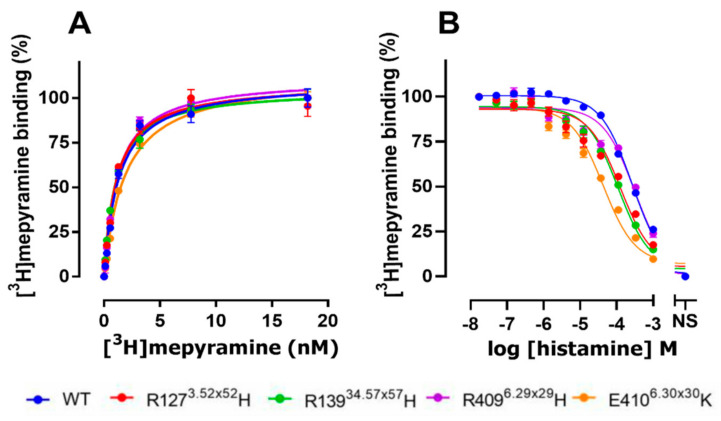
Ligand binding to hH_1_R variants. (**A**) Specific binding of increasing concentrations [^3^H]mepyramine to HEK293T cell homogenates expressing hH_1_R variants is shown as percentage specific binding of 18 nM [^3^H]mepyramine. Data are shown as mean ± SEM Figure 3. independent experiments performed in duplicate. (**B**) Competition binding between 7 nM [^3^H]mepyramine and increasing histamine concentrations. Data are shown as mean ± SEM from 4 independent experiments performed in triplicate. Nonspecific (NS) binding was determined in the presence of 10 μM mianserin.

Initially, the binding characteristics of the four selected natural hH_1_R variants were investigated. Therefore, the various hH_1_R variants were transiently expressed in HEK293T cells and subjected to [^3^H]mepyramine radioligand binding studies. In saturation binding studies, the antagonist radioligand [^3^H]mepyramine displays a similar nanomolar binding affinity for the naturally occurring hH_1_R variants R127^3.52×52^H, R139^34.57×57^H, R409^6.29×29^H, and E410^6.30×30^K as compared to wild-type (WT) hH_1_R (Figure 4A and Table 3; *p* > 0.05, one-way ANOVA with Dunnett’s multiple comparisons). Moreover, all variants were also well expressed, as indicated by the high B_max_ values following [^3^H]mepyramine saturation binding (Table 3). Next, [^3^H]mepyramine competition binding studies were conducted to determine the affinity of the unlabeled antagonists, mepyramine, levocetirizine, and doxepin and the endogenous agonist histamine. The three tested antagonists all have similar binding affinities for all four variants as compared to WT hH_1_R (Table 4). In contrast, the agonist histamine displays a 6.3-fold increased binding affinity for the E410^6.30×30^K variant compared to WT hH_1_R (Figure 4B; Table 4). A smaller increase in histamine affinity was observed for R127^3.52×52^H and R139^34.57×57^H (2.5- and 3.1-fold, respectively), whereas R409^6.29×29^H has the same affinity as WT hH_1_R.

### 2.3. The E410^6.30×30^K Variant Displays Increased Constitutive Activity in G Protein Signaling

**Figure 5:**
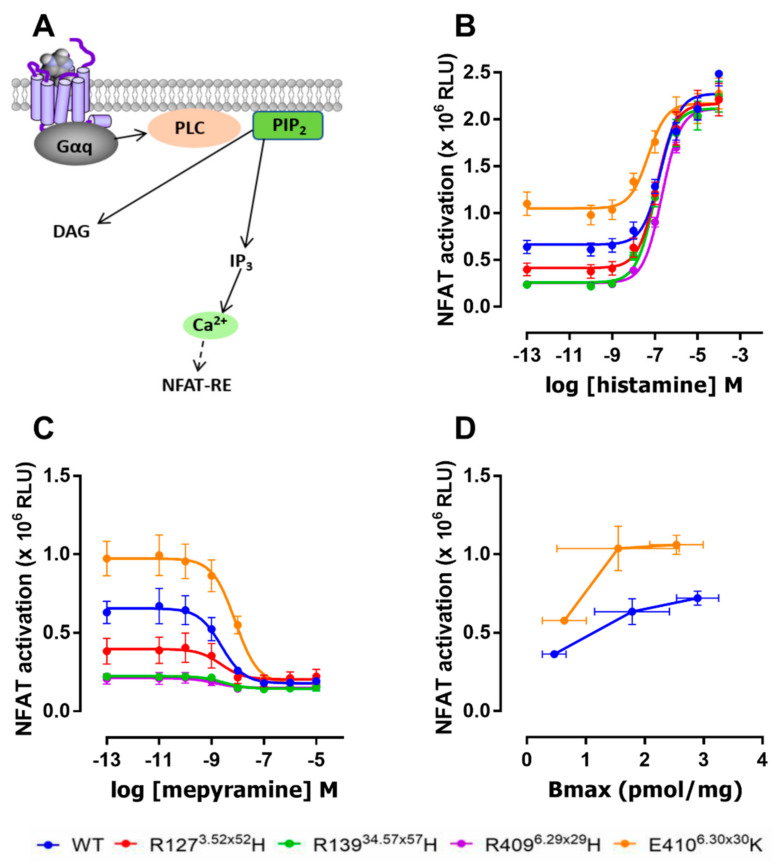
G_q_ protein signaling by hH_1_R variants. (**A**) G_q_-mediated activation of phospholipase C (PLC) resulting in the cleavage of phosphatidylinositol 4,5-bisphosphate (PIP_2_) into diacylglycerol (DAG) and inositol 1,4,5-trisphosphate (IP_3_), and the subsequent increase in intracellular Ca^2+^ levels are measured as NFAT-driven luciferase reporter gene expression (NFAT-RE). (**B**,**C**) HEK293T cells co-transfected with hH_1_R variants and NFAT-reporter gene plasmids were incubated with increasing histamine concentrations (**B**) or inverse agonist mepyramine (**C**). Data are shown in relative light units (RLU) as mean ± SEM from 3 independent experiments performed in duplicate. (**D**) NFAT-driven reporter gene activity in HEK293T cells expressing increasing levels of the constitutively active WT and E410^6.30×30^K hH_1_R variants. Receptor expression was measured by [^3^H]mepyramine binding (B_max_). Data are shown as mean ± SEM from 4 independent experiments performed in triplicate.

To evaluate the effect of the naturally occurring R127^3.52×52^H, R139^34.57×57^H, R409^6.29×29^H, and E410^6.30×30^K variants on G protein-mediated hH_1_R signaling, we measured hH_1_R-mediated NFAT-driven luciferase activity in response to histamine stimulation (Figure 5A). The E410^6.30×30^K variant showed increased basal signaling as compared to WT hH_1_R in HEK293T cells transiently transfected with 5 ng receptor-encoding DNA per dish, whereas R127^3.52×52^H, R139^34.57×57^H, and R409^6.29×29^H showed reduced constitutive activity (Figure 5B). Histamine induces NFAT reporter gene activity in cells expressing WT hH_1_R with a pEC_50_ of 6.7 ± 0.05 (*n* = 3, Figure 5B; Table 5), as previously observed in this reporter gene readout [27]. Histamine has the same potency in cells expressing the R127^3.52×52^H, R139^34.57×57^H, and R409^6.29×29^H variants as compared to WT hH_1_R, whereas significantly higher potency (0.6 log unit, 4-fold) was observed in cells expressing the E410^6.30×30^K variant (Figure 5B; Table 5).

To confirm the observed effects of these natural variants on the hH_1_R constitutive activity in the NFAT-driven reporter gene assay, transfected HEK293T cells were incubated with the hH_1_R inverse agonist mepyramine [21]. Indeed, mepyramine concentration-dependently inhibited basal signaling of all hH_1_R variants with comparable potencies for R127^3.52×52^H, R139^34.57×57^H, R409^6.29×29^H, and WT hH_1_R (Figure 5C, Table 5). However, mepyramine displays a 4-fold (0.6 log unit) reduced potency to inhibit E410^6.30×30^K constitutive signaling to NFAT, as compared to WT hH_1_R (Figure 5C; Table 5). To further confirm that E410^6.30×30^K displayed increased constitutive activity as compared to WT, HEK293T cells were co-transfected with the NFAT-driven reporter gene plasmid in combination with 1, 5, and 10 ng of WT hH_1_R or E410^6.30×30^K encoding plasmids. Indeed, at comparable expression (B_max_) levels, E410^6.30×30^K displays a higher level of constitutive activity than WT hH_1_R (Figure 5D).

## 3. Discussion

Despite the increasing accumulation of data on genetic variation in protein-coding sequences in the human population through international consortia, such as the ExAC, a huge gap still exists between the predicted effect of these natural amino acid substitutions based on bioinformatic SIFT and/or PolyPhen scores, and the experimental evaluation of their functional impact on protein function. Recent pharmacogenomic analysis of this ExAC dataset on approved GPCR drug targets revealed that 9522 out of the 14,192 MVs are predicted to be functionally deleterious based on their SIFT or PolyPhen scores, and 1772 MVs involve amino acids in the ligand-binding pocket, conformational microswitches, and G protein/arrestin interaction interface [5].

In this study, we focused this analysis on the four members of the histamine receptor family, showing that 129 predicted deleterious MVs (45% and 28% of deleterious and total MVs, respectively) are mapped to these functional GPCR domains, and consequently might likely affect histamine receptor function. Indeed, our experimental analysis shows that the E410^6.30×30^K MV in the microswitch domain of H_1_R displays increased affinity for the endogenous agonist histamine in combination with increased constitutive activity in G_q_ protein signaling, suggesting that this H_1_R variant adopts a more active conformational state as compared to WT H_1_R. This indicates that E410^6.30×30^ in wild-type H_1_R is most likely involved in biasing the conformational ensemble of H_1_R towards the inactive state, probably by forming an ionic lock with the D(E)RY motif in TM3 as reported for several other GPCRs [15,16,17,18]. The absence of this ionic lock interaction in the crystal structure of doxepin-bound inactive H_1_R might be the consequence of the replacement of ICL3 with the T4 lysozyme [20], as also observed in other crystal structures of inactive GPCR-T4 lysozyme fusions [28]. Indeed, mutations of E6.30 in other GPCR subtypes are also reported to affect ligand affinity and receptor activity. To illustrate, mutations of this residue to electrostatically neutral or positively-charged residues commonly show increased binding affinity for agonists, as well as an increase in basal activity for a variety of receptors that include the muscarinic acetylcholine m1 and m2 receptor [29,30], β_2_ adrenergic receptor [17,31], 5-hydroxytryptamine receptor 2A [32], and α_1B_ adrenergic receptor [33]. Moreover, natural occurring D6.30 MVs in the follicle-stimulating hormone receptor, luteinizing hormone receptor, and thyroid-stimulating hormone receptor also display increased constitutive receptor activity and are consequently associated with familial spontaneous ovarian hyperstimulation syndrome [34], familial male-limited precocious puberty [35], and hyperfunctioning thyroid adenomas [36], respectively. Interestingly, the ExAC dataset also revealed E229^6.30×30^K in the H_2_R as a possible deleterious MV. Consequently, the functional effect of this MV might be further investigated in the future, especially since mutation of the DRY motif in the H_2_R has been shown to increase agonist binding affinity, constitutive activity, and structural H_2_R instability [19]. These experimental observations nicely fit with the hypothesis of an ionic lock in the inactive state of the H_2_R, and the E229^6.30×30^ K MV would, therefore, also expected to increase constitutive H_2_R activity.

The doxepin bound H_1_R structure reveals three positively charged amino acids at an interacting distance to the D(E)RY motif and ionic lock counterpart (E410^6.30×30^K), which have been reported to carry rare, but potentially deleterious MVs [20]. We hypothesized that given the 3D spatial proximity and their charge, these residues most likely are involved in the intricate network of the ionic lock and hence affect receptor pharmacology when mutated. This hypothesis is supported by experimental evidence obtained for other receptors where residues in the proximity to the ionic lock stabilize or destabilize this interaction, such as arginine in the ICL2 of the constitutively active viral GPCR US28, which destabilizes the inactive D(E)RY conformation [37]. Indeed, our experimental analysis shows that MVs R127^3.52×52^H, R139^34.57×57^H, R409^6.29×29^H reduce receptor-constitutive activity compared to WT. The R^6.29×29^ is conserved among all 4 histamine receptor subtypes (Supplementary Figure S2), and the R297^6.29×29^G MV was identified for the H_4_R in the ExAc dataset. The effect of this MV on H_4_R constitutive signaling remains to be investigated. Amino acids at positions 3.52 × 52 and 34.57 × 57 are not conserved between the four histamine subtypes (Supplementary Figure S2).

Although single-nucleotide polymorphisms (SNPs) in histamine receptor genes have been reported in the literature in relation to CNS disorders, inflammation, and cancer, and showing effects of SNPs in the non-coding gene sequences (UTRs and introns), only a few studies have hitherto investigated the impact of MVs in relation to these diseases [38,39]. The impact of H_1_R MVs K19N, D349E (ICL3), Q356H (ICL3), and L449^7.34×33^S on atypical antipsychotic-induced weight gain was investigated as this is observed in many but not all schizophrenia patients. However, these 4 MVs did not affect olanzapine-induced weight gain in both schizophrenia patients and healthy controls after 6 weeks of treatment [40]. Also, longer treatment periods (2–6 months) with clozapine or olanzapine revealed no association between H_1_R MVs D349E (ICL3) and L449^7.34×33^S and weight gain [41,42]. In addition, the L449^7.34×33^S MV could not be associated with Parkinson’s disease [43]. The D349A (ICL3) MV did not affect aspirin-induced urticarial/angioedema in a Korean population. Previous analysis of the Ensembl (http://www.ensembl.org (accessed on 20 January 2021)) and NCBI SNPdb (http://www.ncbi.nlm.nih.gov/snp (accessed on 20 January 2021)) databases in a genomic study on the predictive role of H_1_R expression in hematological and solid tumors revealed 84 H_1_R MVs among the 2455 available single nucleotide polymorphisms (SNPs) [44]. However, the functional impact of these MVs and their direct association with cancer was not further investigated in this study. To our best knowledge, none of the identified H_2_R and H_4_R MVs have so far been reported to have an impact on diseases. Interestingly, the H_3_R MV A280V (ICL3) was first observed in a patient with Shy-Drager syndrome but later identified as a risk factor for migraine in a Mexican population [45,46]. In transfected cells, A280V displayed a reduction in signaling efficacy as compared to wild-type H_3_R without affecting ligand-binding affinities [47].

Hence, these observations highlight the importance of analyzing the genetic variation landscape to better understand receptor function. Our study reveals that missense variations in GPCRs, and specifically in H_1_R, predicted to be deleterious occur on residues involved in key microswitches and functional domains, which are indeed likely to affect the pharmacology of the receptor. However, this relationship is not often validated. Our analysis shows that the E410^6.30×30^K H_1_R variant disrupts the ionic lock microswitch with the D(E)RY motif in TM3 that restrains the H_1_R in a more inactive conformation resulting in increased constitutive activity and histamine affinity. Our results highlight how missense variants can result in receptors that behave pharmacologically different from the wild-type receptor and hence should be considered in the drug discovery process. Moreover, as discussed in detail by Hauser et al., the impact that these genetic variations have on GPCR pharmacology plays a role in human disease directly or indirectly [5]. Directly because genetic variations can induce various degrees of abnormal receptor (de)activation, from pathologically inactive to hyperactive receptors [48,49]. This is illustrated by mutant GPCRs in diseases, such as melanocortin receptors in obesity, extracellular calcium-sensing (CAS) receptor in hypocalcemia, and many others. Furthermore, GPCR genetic variations can also affect human health in an indirect way, as patients might respond differently to therapeutics that target these GPCR variants due to changed receptor pharmacology [5].

## 4. Materials and Methods

### 4.1. Materials

[^3^H]mepyramine (specific activity 20.7 Ci/mmol), MicroScint-O scintillation liquid, and GF/C filter plates were bought from PerkinElmer (Waltham, MA, USA). Doxepin, mepyramine maleate, levocetirizine·2HCl and histamine·2HCl were obtained from Sigma-Aldrich (St. Louis, MO, USA). NanoGlo^®^ was bought from Promega (Madison, WI, USA). Fetal bovine serum (FBS) was obtained from Bodinco (Alkmaar, The Netherlands), and penicillin/streptomycin was purchased from GE Healthcare (Uppsala, Sweden). FastDigest^TM^ restriction enzymes, Dulbecco’s modified Eagles medium (DMEM), Dulbecco’s phosphate-buffered saline (DPBS), trypsin-EDTA, Hanks’ balanced salt solution (HBSS), Pierce^TM^ BCA protein assay kit, GeneJET gel extraction kit and GeneJET plasmid Miniprep kit were bought from Thermo Fisher Scientific (Waltham, MA, USA). 25 kDa linear polyethylenimine (PEI) was purchased from Polysciences (Warrington, PA, USA). The Nluc-hH_1_R/pcDNA3.1 construct was previously reported [50]. The reporter gene construct pNFAT-luc was obtained from Agilent Technologies (Santa Clara, CA, USA). All other reagents were of analytical grade and obtained from conventional commercial sources.

### 4.2. Residue Numbering

To allow systematic comparison of the amino acid residues at different positions in different GPCRs, receptor residue numbers are annotated throughout this study by their Uniprot numbers (for specific receptors only) complemented by their Ballesteros–Weinstein residue number and secondary structure motif in superscript [51,52]. According to the Ballesteros–Weinstein GPCR residue numbering schemes, the most conserved residue in each TM helix is designated X.50. For the most conserved loops, similar residue numbering schemes have been applied. For example, ECL2 residues are labeled 45.X, and the reference residue C^45.50^ is a conserved cysteine forming a disulfide bridge with C^3.25^ in TM3 [53].

### 4.3. Genetic Variation Dataset

Genetic variation data for GPCRs were retrieved from the genetic receptor variants website on GPCRdb (https://gpcrdb.org/mutational_landscape/; accessed on 20 January 2021) [5,8], which compiles GPCR exome sequences from 60706 unrelated humans from 6 distinct populations that were collected by the Exome Aggregation Consortium (ExAC) [4]. The structural segments of the four histamine receptor subtypes that consist of the N- and C-terminal tail, 7 transmembrane helices, 3 extracellular loops, 3 intracellular loops, and helix8 were assigned based on GPCRdb (https://gpcrdb.org/protein/; accessed on 20 January 2021), as previously described [5,8]. The missense variants (MV) were projected onto each structural segment, and MV density was subsequently calculated by normalizing for the segment length, as previously described [5]. The minor allele frequencies (MAF) of MVs are reported on GPCRdb and represent the allele counts of the less frequent allele divided by the total number of alleles at that locus in the ExAC dataset (https://gpcrdb.org/mutational_landscape/; accessed on 20 January 2021) [5,8]. MAF scores < 1 × 10^−3^ and ≥ 1 × 10^−3^ indicate rare and common MVs, respectively. The impact of MVs was classified as functionally tolerated or deleterious by their SIFT and PolyPhen scores that are reported on GPCRdb (https://gpcrdb.org/mutational_landscape/; accessed on 20 January 2021) [5,8]. The SIFT (sorting intolerant from tolerant) scores are from 0–1 with 0–0.05 being deleterious and >0.05 being tolerated [54], whereas PolyPhen scores are from 0–1 with 0–0.1 being tolerated and > 0.1 being deleterious [55].

The tolerated and deleterious MVs in the histamine receptor family were cross-mapped with amino acid residue positions that are (putatively) involved in ligand entry (i.e., extracellular vestibule), orthosteric ligand binding, conformational microswitches, and GPCR-G protein/arrestin interaction interface, essentially as previously described [5]. The extracellular vestibule is defined as the amino acid residues in the N-terminal tail, extracellular loops (ECL) 1, 2, and 3. Amino acid positions that align the orthosteric ligand binding site in the histamine receptor family were derived from the doxepin-bound H_1_R crystal structure and site-directed mutagenesis data reported in the mutant browser on GPCRdb (https://gpcrdb.org/mutations/; accessed on 20 January 2021), and consist of: 2.61 × 60, 2.66 × 65, 3.28 × 28, 3.32 × 32, 3.33 × 33, 3.36 × 36, 3.37 × 37, 3.40 × 40, 4.51 × 51, 4.57 × 57, 4.56 × 57, 5.36 × 37, 5.39 × 40, 5.41 × 42, 5.42 × 43, 5.44 × 45, 5.46 × 461, 5.47 × 47, 5.48 × 48, 6.44 × 44, 6.48 × 48, 6.51 × 51, 6.52 × 52, 6.55 × 55, 7.42 × 41, and 7.43 × 42 [8,20]. Reported amino acids positions that are involved in conformational GPCR microswitches include the ionic lock: 3.49 × 49, 3.50 × 50, 3.51 × 51, and 6.30 × 30; the NPxxY motif: 7.49 × 49, 7.50 × 50, and 7.53 × 53; the CWxP motif: 6.47 × 47, 6.48 × 48, and 6.50 × 50; the transmission switch, also known as PIF motif: 3.40 × 40, 5.50 × 50, and 6.44 × 44; and the allosteric sodium pocket: 2.50 × 50, 3.39 × 39, 6.48 × 48, 7.45 × 45, and 7.46 × 46 [56,57,58,59]. Amino acid positions that putatively form the GPCR-G protein/arrestin interaction interface were assigned based on contacts observed in crystal structures of GPCRs in complex with G protein or arrestin and consist of: 12.48 × 48, 2.36 × 36, 2.37 × 37, 2.39 × 39, 2.40 × 40, 3.49 × 49, 3.50 × 50, 3.53 × 53, 3.54 × 54, 3.55 × 55, 3.56 × 56, 34.50, 34.51 × 51, 34.52 × 52, 34.53 × 53, 34.54 × 54, 34.55 × 55, 34.57 × 57, 34.58 × 58, 4.36 × 36, 4.38 × 38, 4.39 × 39, 4.40 × 40, 4.41 × 41, 5.58 × 58, 5.61 × 61, 5.64 × 64, 5.65 × 65, 5.67 × 67, 5.68 × 68, 5.69 × 69, 5.71 × 71, 5.72 × 72, 5.74 × 74, 5.75 × 75, 5.76 × 76, 6.22 × 22, 6.23 × 23, 6.24 × 24, 6.25 × 25, 6.26 × 26, 6.28 × 28, 6.29 × 29, 6.30 × 30, 6.32 × 32, 6.33 × 33, 6.36 × 36, 6.37 × 37, 6.40 × 40, 7.55 × 55, 7.56 × 56, 8.47 × 47, 8.48 × 48, 8.49 × 49, 8.51 × 51, and 8.52 × 52 [8,60,61,62,63,64].

### 4.4. Generation of hH_1_R Variants

The hH_1_R variants R127^3.52×52^H, R139^34.57×57^H, R409^6.29×29^H, and E410^6.30×30^K, were created by PCR-based mutagenesis using wild-type hH_1_R (GenBank: NM_00861) as template [65]. The PCR fragments were subcloned into Nluc-hH_1_R /pcDNA3.1 plasmid using the internal restriction sites PflMI and EcoRI, and subsequently sequence verified at Eurofins Genomics (Ebersberg, Germany).

### 4.5. Cell Culture and Transfection

HEK293T cells (ATCC; Manassas, VA, USA) were maintained at 37 °C, 5% CO_2_ in culture medium (Dulbecco’s modified Eagle’s medium (DMEM) supplemented with 10% FBS and 1% penicillin/streptomycin)_._ Transfections of HEK293T cells were performed using the PEI method, as previously described [27]. Briefly, 2 × 10^6^ HEK293T cells were seeded in a 10 cm dish. The next day, 5 μg DNA plasmid is mixed by vortexing with 20 μg of linear PEI (1:4 ratio) in 150 mM NaCl solution and incubated for 30 min at room temperature. Empty pcDEF3 plasmid was used to keep total DNA equal. The transfection mix is gently resuspended and added dropwise to one dish with cells containing 6 mL fresh medium after incubation.

### 4.6. Radioligand Binding Experiments

HEK293T cells were collected two days after transfection with 1 µg of Nluc-hH_1_R variant-encoding plasmids per dish and homogenized in binding buffer (50 mM Na_2_HPO_4_/KH_2_PO_4_, pH 7.4) as previously described [27]. For saturation binding assay, cell homogenates were incubated with increasing concentrations of [^3^H]mepyramine (0–20 nM) for 1 hour at 25 °C with gentle agitation in the absence or presence of 10 µM mianserin to detect total and nonspecific binding, respectively. For competition binding assay, cell homogenates were incubated with 7 nM [^3^H]mepyramine in combination with increasing concentrations of unlabeled histamine, doxepin, mepyramine, or levocetirizine, for 1 hour at 25 °C with gentle agitation. Incubations were stopped by rapid filtration with ice-cold wash buffer (50 mM Tris-HCl, pH 7.4) over a 0.5% polyethyleneimine-coated 96-well GF/C filter plate using a 96-well FilterMate-harvester (Perkin-Elmer; Waltham, MA, USA). The GF/C filter plates were dried, and filter-bound radioactivity was quantified after the addition of 25 μL/well MicroScint-O using a Wallac 1450 MicroBeta Trilux counter (PerkinElmer). The binding affinity (K_d_) of [^3^H]mepyramine and the total number of receptors (B_max_) were determined using the “One site—Total and nonspecific binding” model in GraphPad Prism 8.4.3. The protein content of the cell homogenates was measured by the BCA kit. Competition binding curves were fitted using the “one-site—Fit logIC50” model in GraphPad Prism 8.4.3, and binding affinities (K_i_) of unlabeled ligands were subsequently calculated using the Cheng–Prusoff equation:

$$K_{i}=\;\frac{IC_{50}}{1+\frac{\left[L\right]}{K_{d}}}$$

where [*L*] and *K_d_* are the concentration and binding affinity of [^3^H]mepyramine, respectively.

### 4.7. Nuclear Factor Activated T-Cells (NFAT)-Driven Reporter Gene Assay

HEK293T cells were transiently co-transfected with 5 ng Nluc-hH_1_R variants and 2.5 µg NFAT-Luc reporter gene plasmids per dish and transferred (50,000 cells/well) into poly-L-lysine-coated white 96-well plates after 24 h, as previously described [27]. The cells were stimulated the next day with histamine for 6 hours to detect agonism, whereas mepyramine was added directly after transferring the cells into the 96-well plates to detect inverse agonism. The incubations were terminated by replacing the medium with 25 µL of luciferase assay reagent (0.83 mM ATP, 0.83 mM d-luciferin,18.7 mM MgCl_2_, 0.78 µM Na_2_HPO_4_, 38.9 mM Tris (pH 7.8), 0.39% (*v/v*) glycerol, 0.03% (*v/v*) Triton X-100, and 2.6 µM dithiothreitol). Luminescence was measured (1 s/well) after another 30 min incubation at 37 °C in a Mithras LB940 multimode microplate reader (Berthold, Germany). Concentration–response curves were fitted using the “log (agonist) vs. response (three parameters)” model in GraphPad Prism 8.4.3.

### 4.8. Data Analysis

GraphPad Prism version 8.4.3 (GraphPad Software, San Diego, CA, USA) was used for nonlinear regression and statistics.

## Figures and Tables

**Table 1:** Missense variations (MV) in G protein-coupled receptors (GPCR) from nearly 61,000 unrelated individuals in the exome aggregation consortium (ExAC) database.

	Total Missense Variations	Average Density ^1^	Total Rare MVs ^2^	Total Common MVs ^3^	Predicted Deleterious Mutations ^4^	Total Loss-of-Function Variants ^5^
All GPCRs ^6^	65,539	0.27	155 ^7^	4.8 ^7^		4066
Class A GPCRs ^8^	36,340	0.27	35,323	1017	24,207	2305
Histamine receptors	463	0.24	453	10	289	30
H_1_R	154	0.32	149	5	86	6
H_2_R	71	0.20	70	1	36	1
H_3_R	117	0.26	117	0	83	2
H_4_R	121	0.31	117	4	84	21

^1^ Absolute count/length of receptor. ^2^ Minor allele frequency (MAF) < 1 × 10^−3^. ^3^ Minor allele frequency (MAF) ≥ 1 × 10^−3^. ^4^ sorting intolerant from tolerant (SIFT) and polymorphism phenotyping (PolyPhen) scoring systems are algorithms that predict the impact of missense variation on protein structure and function. SIFT ≤ 0.05 or PolyPhen ≥ 0.1 is considered deleterious. ^5^ Loss of function mutations are frame-shift mutation or introduced stop codon. ^6^ All 401 non-olfactory GPCRs. ^7^ Average per receptor. ^8^ 290 class A GPCRs.

**Table 2:** ExAC data extracted from the GPCRdb ^1^ for the rare, deleterious H_1_R MVs selected in this study.

Position	GPCRdb	Segment	Variant	Allele Count ^2^	Allele Number ^3^	Allele Frequency ^4^	Homozygotes ^5^	SIFT ^6^	PolyPhen ^7^
127	3.52 × 52	TM3	R => H	3	121266	2.47 × 10^−5^	0	0.02	0.909
139	34.57 × 57	ICL2	R => H	7	121276	5.77 × 10^−5^	1	0	1
409	6.29 × 29	TM6	R => H	2	121228	1.65 × 10^−5^	0	0	0.996
410	6.30 × 30	TM6	E => K	4	121242	3.30 × 10^−5^	0	0	0.996

^1^https://gpcrdb.org/mutational_landscape/protein/hrh1_human/ (accessed on 20 January 2021). ^2^ Number of alleles with this variant in ExAC dataset. ^3^ Total number of alleles analyzed in ExAC dataset. ^4^ Minor allele frequency calculated as alleles with variant/total number of alleles. Frequency < 1 × 10^−3^ is considered rare. ^5^ Number of individuals in ExAC dataset with both alleles affected (=homozygotes). ^6^ SIFT ≤ 0.05 is considered deleterious. ^7^ PolyPhen ≥ 0.1 is considered deleterious.

**Table 3:** Binding affinity (pK_d_) of [^3^H]mepyramine for hH_1_R variants and their expression levels (B_max_) in HEK293T cells. Data represent the mean ± standard deviation from 3 independent experiments performed in duplicate.

hH_1_R Variants	pK_d_	B_max_ (pmol/mg)
WT	8.9 ± 0.10	42.5 ± 12.07
R127^3.52×52^H	8.9 ± 0.13	35.1 ± 12.01
R139^34.57×57^H	9.0 ± 0.06	38.4 ± 8.42
R409^6.29×29^H	8.9 ± 0.03	48.4 ± 12.33
E410^6.30×30^K	8.7 ± 0.05	27.8 ± 7.39

**Table 4:** Binding affinity (pK_i_) of histamine and three representative H_1_ antagonists for the hH_1_R variants. Data represent the mean ± standard deviation from at least 3 independent experiments performed in duplicate. Statistical differences (*p* < 0.05) compared to WT were determined using one-way ANOVA with Dunnett’s multiple comparison test and are indicated by an asterisk (*).

hH_1_R Variants	Histamine	Mepyramine	Levocetirizine	Doxepin
WT	4.3 ± 0.05	8.8 ± 0.06	7.7 ± 0.17	9.7 ± 0.19
R127^3.52×52^H	4.7 ± 0.02 *	8.9 ± 0.09	7.6 ± 0.08	9.8 ± 0.17
R139^34.57×57^H	4.8 ± 0.03 *	8.8 ± 0.09	7.8 ± 0.09	9.6 ± 0.17
R409^6.29×29^H	4.3 ± 0.09	9.0 ± 0.33	7.8 ± 0.17	9.9 ± 0.29
E410^6.30×30^K	5.1 ± 0.10 *	8.7 ± 0.16	7.8 ± 0.17	9.7 ± 0.06

**Table 5:** Potency of histamine (pEC_50_) and mepyramine (pIC_50_) to induce or inhibit nuclear factor activated t-cells (NFAT) reporter gene activation, respectively, in HEK293T cells expressing the hH_1_R variants. Data represent the mean ± standard deviation from 3 independent experiments performed in duplicate. Statistical differences (*p* < 0.05) compared to WT were determined using one-way ANOVA with Dunnett’s multiple comparison test and are indicated by an asterisk (*).

hH_1_R Variants	Histamine	Mepyramine
WT	6.7 ± 0.05	8.7 ± 0.23
R127^3.52×52^H	6.9 ± 0.09	8.6 ± 0.20
R139^34.57×57^H	7.0 ± 0.06	8.6 ± 0.13
R409^6.29×29^H	6.7 ± 0.09	8.8 ± 0.27
E410^6.30×30^K	7.3 ± 0.23 *	8.1 ± 0.22 *

## Data Availability

Not applicable.

## Supplementary Materials

The following are available online at https://www.mdpi.com/article/10.3390/ijms22073702/s1, Figure S1: Frequency and distribution of MVs in histamine receptor family. The number of rare and common MVs was determined for the four histamine receptor subtypes, and their structural segments rare, and common MVs were determined by their reported minor allele frequency scores of <1 × 10^−3^ and ≥1 × 10^−3^, respectively. Figure S2: Location of missense variants in the histamine receptor family. Snake plots for the H_1_R, H_2_R, H_3_R, and H_4_R were extracted from GPCRdb and tolerated, and deleterious MVs as predicted by SIFT/PolyPhen analyses are highlighted in green and red, respectively.
