# Supplementary material for: Analysis of Missense Variants in the Human Histamine Receptor Family Reveals Increased Constitutive Activity of E4106.30×30K Variant in the Histamine H1 Receptor
Source: Int J Mol Sci. 2021 Apr 2;22(7):3702. doi: 10.3390/ijms22073702 (PMC8038156; doi:10.3390/ijms22073702)
Supplement: Supplementary file 1 [file ijms-22-03702-s001.pdf]

Analysis of missense variants in the human histamine receptor family reveals increased constitutive activity of E410$^{6.30x30}$K variant in the histamine H_1 receptor

Xiaoyuan Ma†, Marta Arimont Segura†, Barbara Zarzycka, Henry F. Vischer, Rob Leurs*.
Amsterdam Institute for Molecules, Medicines and Systems, Division of Medicinal Chemistry, Faculty of Science, Vrije Universiteit Amsterdam, Amsterdam, The Netherlands.

SUPPLEMENTARY MATERIALS

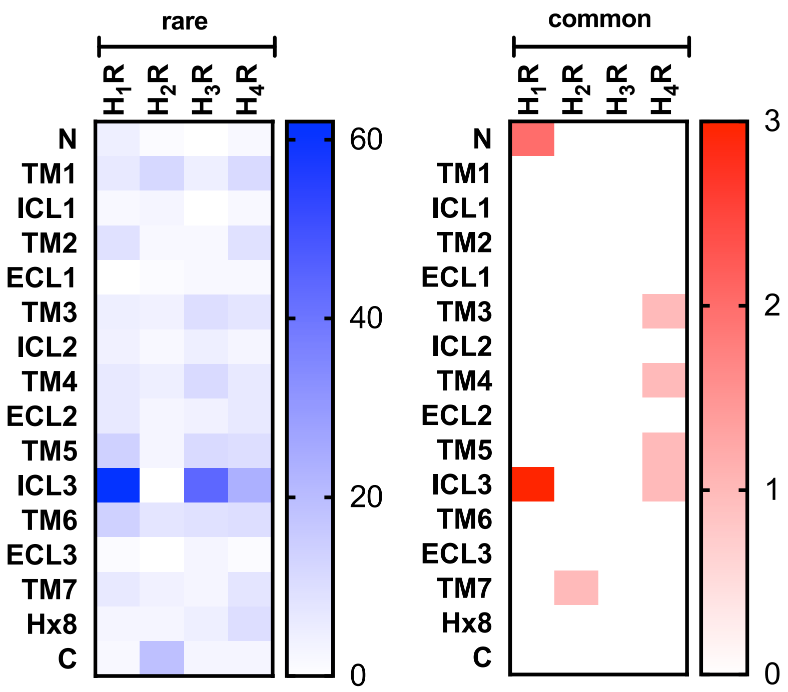

Supplementary Figure S1. Frequency and distribution of MVs in histamine receptor family. Number of rare and common MVs was determined for the four histamine receptor subtypes and their structural segments rare and common MVs were determined by their reported minor allele frequency scores of $<1x10^{-3}$ and $\geq 1x10^{-3}$, respectively.

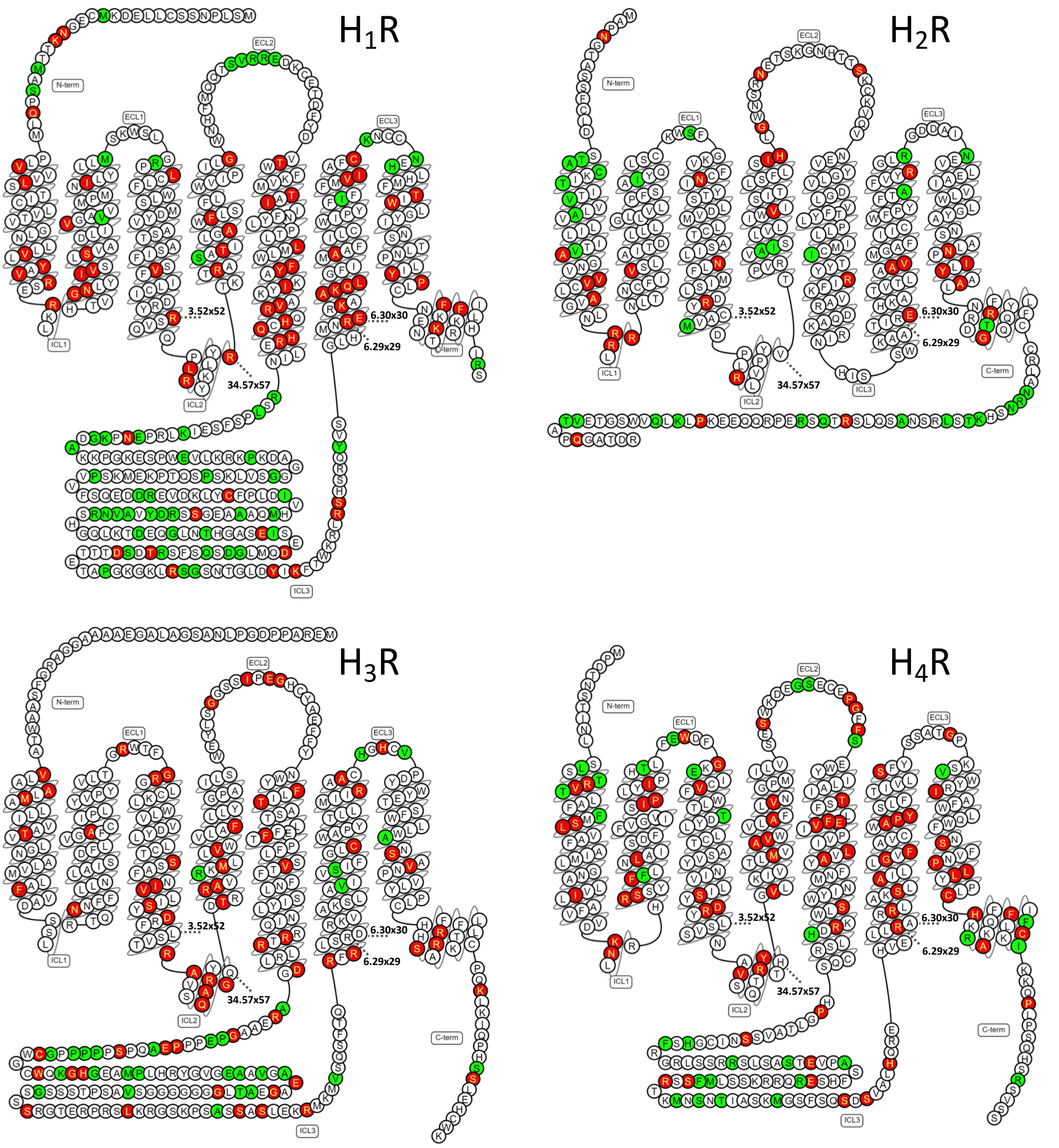

Supplementary Figure S2. Location of missense variants in the histamine receptor family. Snake plots for the H_1R, H_2R, H_3R, and H_4R were extracted from GPCRdb (https://gpcrdb.org/mutational_landscape/statistics) and tolerated and deleterious MVs as predicted by SIFT/PolyPhen analyses are highlighted in green and red, respectively. The positions of the four H_1R missense variants that were experimentally analysed are indicated with their generic numbers and corresponding amino acids at these positions are indicated in the H_2R, H_3R, and H_4R.
